# An empirical study of ensemble-based semi-supervised learning approaches for imbalanced splice site datasets

**DOI:** 10.1186/1752-0509-9-S5-S1

**Published:** 2015-09-01

**Authors:** Ana Stanescu, Doina Caragea

**Affiliations:** 1 Department of Computing and Information Sciences, Kansas State University, Nichols Hall, Manhattan, KS, 66506, USA

**Keywords:** acceptor splice sites, semi-supervised learning, self-training, co-training, imbalanced data, ensemble learning

## Abstract

**Background:**

Recent biochemical advances have led to inexpensive, time-efficient production of massive volumes of raw genomic data. Traditional machine learning approaches to genome annotation typically rely on large amounts of labeled data. The process of labeling data can be expensive, as it requires domain knowledge and expert involvement. Semi-supervised learning approaches that can make use of unlabeled data, in addition to small amounts of labeled data, can help reduce the costs associated with labeling. In this context, we focus on the problem of predicting splice sites in a genome using semi-supervised learning approaches. This is a challenging problem, due to the highly imbalanced distribution of the data, *i.e*., small number of splice sites as compared to the number of non-splice sites. To address this challenge, we propose to use ensembles of semi-supervised classifiers, specifically self-training and co-training classifiers.

**Results:**

Our experiments on five highly imbalanced splice site datasets, with positive to negative ratios of 1-to-99, showed that the ensemble-based semi-supervised approaches represent a good choice, even when the amount of labeled data consists of less than 1% of all training data. In particular, we found that ensembles of co-training and self-training classifiers that dynamically balance the set of labeled instances during the semi-supervised iterations show improvements over the corresponding supervised ensemble baselines.

**Conclusions:**

In the presence of limited amounts of labeled data, ensemble-based semi-supervised approaches can successfully leverage the unlabeled data to enhance supervised ensembles learned from highly imbalanced data distributions. Given that such distributions are common for many biological sequence classification problems, our work can be seen as a stepping stone towards more sophisticated ensemble-based approaches to biological sequence annotation in a semi-supervised framework.

## Background

Advances in biochemical technologies over the past decades have given rise to Next Generation Sequencing platforms that quickly produce genomic data at much lower costs than ever before. Such overwhelmingly large volumes of sequenced DNA remain difficult to annotate. As a result, numerous computational methods for genome annotation have emerged, including machine learning and statistical analysis approaches that practically and efficiently analyze and interpret data. Supervised machine learning algorithms typically perform well when large amounts of labeled data are available. In bioinformatics and many other data-rich disciplines, the process of labeling instances is costly; however, unlabeled instances are inexpensive and readily available. For a scenario in which the amount of labeled data is relatively small and the amount of unlabeled data is substantially larger, semi-supervised learning represents a cost-effective alternative to manual labeling.

Because semi-supervised learning algorithms use both labeled and unlabeled instances in the training process, they can produce classifiers that achieve better performance than completely supervised learning algorithms that have only a small amount of labeled data available for training [1-3]. The principle behind semi-supervised learning is that intrinsic knowledge within unlabeled data can be lever-aged in order to strengthen the prediction capability of a supervised model that only uses labeled instances, thereby providing a potential advantage for semi-supervised learning. Model parameters learned by a supervised classifier from a small amount of labeled data may be steered towards a more realistic distribution (which more closely resembles the distribution of the test data) by the unlabeled data.

Unfortunately, unlabeled data can also drive the model parameters away from the true distribution if misclassification errors reinforce themselves. Thus, in practice, semi-supervised learning does not always work as intended [4-6]. Moreover, under incorrect assumptions, *e.g.*, regarding the relationship between marginal and conditional distributions of data, semi-supervised learning models risk to perform worse than their supervised counterparts. Given that for many prediction problems the assumptions made by learning algorithms cannot be easily verified without considerable domain knowledge [7] or data exploration, semi-supervised learning is not always "safe" to use. Advantageous utilization of the unlabeled data is problem-dependent, and more research is needed to identify algorithms that can be used to increase the effectiveness of semi-supervised learning [8,9], in general, and for bioinformatics problems, in particular. At a high level, we aim to identify semi-supervised algorithms that can be used to learn effective classifiers for genome annotation tasks.

In this context, a specific challenge that we address is the "data imbalance" problem, which is prevalent in many domains, including bioinformatics. The data imbalance phenomenon arises when one of the classes to be predicted is underrepresented in the data because instances belonging to that class are rare (noteworthy cases) or hard to obtain. Ironically, minority classes are typically the most important to learn, because they may be associated with special cases. In general, anomaly or novelty detection problems exhibit highly imbalanced distributions. Specific applications outside the bioinformatics area include credit card fraud, cyber intrusions, medical diagnosis, face recognition, defect detection in error-prone software modules, *etc*. As established in the literature (*e.g*., [10]), the existence of a major unevenness between the prior class probabilities leads to impartial learning. As a result, classifiers that produce good classification results under normal circumstances (*i.e*., in the presence of balanced or mildly imbalanced distributions) can be seriously compromised when faced with skewed distributions, as classifiers become strongly biased towards the majority class. In bioinformatics, problems such as promoter recognition, splice site detection, and protein classification are especially difficult because these problems naturally exhibit highly imbalanced distributions.

Resampling datasets in order to reach balanced distributions is a common practice that sometimes improves classification performance, as the model encounters an equal number of instances from each class, thereby producing a more appropriate discriminative function as opposed to a function obtained from skewed distributions. However, it is not well understood what is the most appropriate balancing method. Context-dependent conclusions are usually driven by empirical observations concerning both the classifier used and the imbalance degree. The most straightforward method is under-sampling, in which instances that belong to the majority class are eliminated until a balanced distribution is reached. As a consequence, information is lost, which is obviously not desirable, given the value of labeled instances, yet this is a good way to speed up the computation. Moreover, studies have shown the effectiveness of under-sampling [11] despite its obvious limitations. Over-sampling is another popular resampling method in which instances of the minority class are generated artificially to counterbalance majority instances. These synthetic instances can potentially improve the classifier, as it gains access to more labeled data. The trade-off between longer computation times associated with larger datasets and better classification performance is usually worthwhile. However, with oversampling, classifiers are prone to overfitting, due to duplicate instances.

An algorithmic approach to handle imbalanced data distributions is based on ensembles of classifiers. Limited amounts of labeled data naturally lead to "weaker" classifiers, but ensembles of "weak" classifiers tend to surpass the performance of any single constituent classifier. Moreover, ensembles typically improve the prediction accuracy obtained from a single classifier by a factor that validates the effort and cost associated with learning multiple models. Intuitively, "bagging" several classifiers leads to better overfitting control, since averaging the high variability of individual classifiers also averages the classifiers' overfitting. The first effective model ensemble surfaced in the mid 1990s [12], under the name "bootstrap aggregating" (bagging), which is a meta-algorithm that performs model averaging over models trained on multiple subsets, *i.e*., bootstrap replicates of the training set. The predictions of the models are combined by voting (in the case of classification) or averaging (in the case of regression) in order to output a single final verdict that reflects the ensemble decision. Originally applied to decision trees, bagging can be used with any classification or regression model and it is especially effective in conjunction with utilization of unstable nonlinear models (*i.e*., a small change in the training set can cause a significant change in the model's learned parameters). Ensembles of classifiers that utilize bagging, boosting, and hybrid-approaches for imbalanced datasets in the supervised framework were reviewed by Galar *et al*. [13].

For a comprehensive survey of data resampling and algorithmic approaches to the imbalanced data problem in the supervised learning framework, the reader is referred to [14]. As opposed to supervised learning, fewer efforts have been aimed at the data imbalance problem in the semi-supervised learning framework, with some notable exceptions. In particular, in a previous study [15], we experimented with data resampling and algorithmic solutions and observed that dynamically balancing the classifiers during the semi-supervised iterations of the algorithm is a useful solution that works better than under- and SMOTE (Synthetic Minority Over-sampling Technique) over-sampling for splice site prediction in the context of single semi-supervised classifiers. We also found that ensembles usually tend to perform better than resampling techniques, except for extreme cases when the imbalance degree is 1-to-99, in which case oversampling performs slightly better than the ensemble-based approach. In a subsequent study [16], we empirically evaluated ensembles of self-training semi-supervised classifiers and found that maintaining diversity during the process of semi-supervised learning is an important requirement for the ensemble. In the current study, we experiment with both self-training and co-training, utilizing a different feature representation than the one we used in [16], to accommodate co-training, which requires two views (representations) of the data.

Similar to our prior work, the current study is performed on the problem of predicting splice sites, a challenging but important task in genome annotation [17]. Splice sites are located at the boundaries between exons and introns. At the 3' end of an intron, the "AG" dimer denotes an acceptor splice site; at the 5' end of the intron, the "GT" dimer denotes a donor splice site. Other non-consensus splice sites exist, but they are not considered in this work. We formulate the task of predicting acceptor splice sites as a binary classification problem in which the positive class represents true acceptor splice sites and the negative class is comprised by decoy "AG" sites. We use five relatively large datasets from five organisms. The distribution of the data (ratio of the size of the minority class to majority class) is very skewed - approximately 1% of "AG" dimers are actually acceptor splice sites.

Among others, Sonnenburg *et al*. [18] previously addressed the splice site prediction problem, in the supervised framework, using Support Vector Machines (SVM) and specialized kernels. As opposed to prior work, in this work, our goal is to investigate ensemble-based semi-supervised learning as a potential solution for splice site prediction and to study the effects of imbalanced distributions on semi-supervised algorithms when labeled data is sparse. Given the large datasets of our case study and the numerous models that needed to be trained to simulate different imbalanced degrees for different ensemble variants, we chose Naïve Bayes as the base classifier in co-training and self-training, because of its computation speed and to avoid tuning hyper-parameters (that many other classifiers require in order to perform well). Although theoretically, the *i.i.d*. assumption (that the observed features are identically and independently distributed) does not hold for many problems (including for the problem studied in this work), generative models such as Naïve Bayes can show superior performance to discriminative models such as SVM, especially when small amounts of labeled data are available [19,20].

The rest of this paper is organized as follows. We continue with a review of related work in the next section, where we also contrast our study with other similar studies. In **Methods**, we describe our approaches, namely the semi-supervised learning ensembles based on self-training and co-training. The **Data **section is dedicated to describing the datasets and the feature representation used with our classifiers. The experimental setting is described in **Experimental setup**, starting with the research questions that motivated the study and continuing with details of the evaluation procedure. We discuss the performance of our approaches in **Results**, and finally, in the **Conclusion **section, we conclude the study and suggest directions for future work.

## Related work

Genome annotation is an ample task that requires machine learning and statistical methods to assist experimental approaches, especially given the large amount of genomic data being generated at unprecedented rates. Supervised machine learning approaches have been widely used in bioinformatics for many tasks, including splice site prediction [18,21-24]. For example, human splice site detection was explored in [25] using SVM classifiers with a Gaussian kernel, and in [21] using a combination of Markov Models and SVM classifiers with polynomial kernels. The work in [22] proposed a Markov Model approach for splice site detection in a human dataset with imbalance degrees of 1-to-96 for acceptors and 1-to-116 for donors.

Semi-supervised learning has generally been used in bioinformatics to solve protein classification problems [26-31], with a few notable exceptions focused on DNA classification [2,3]. A small number of studies [32,26,33] have explored the data imbalance problem in the semi-supervised context and proposed effective solutions, but the imbalance degrees were moderate. For example, in [32], the authors addressed the problem of molecule activity prediction and experimented with transductive SVM classifiers on datasets with relatively small sizes (3K instances), exhibiting imbalance degrees no higher than 1-to-40.

As opposed to that, we focus on datasets with higher degrees of imbalance (up to 1-to-99) and study the behavior of semi-supervised learning algorithms when the available labeled data is less than 1% of the total amount of training data. In general, such a small amount of labeled data is expected to lead to weak classifiers, but an ensemble of classifiers could help overcome this shortcoming to some extent. Galar *et al*. [13] showed that, in supervised frameworks, ensembles perform better than single learners trained on resampled data. Lusa and Blagus [34] found that balancing the class prior in the training set via "multiple down-sizing", in other words, training an ensemble of subclassifiers on balanced subsets, is particularly useful for high-dimensional representations. They showed this using a simulated set and a genuine, publicly available dataset from a breast cancer gene expression microarray study. Another study by Li *et al*. [11] also concluded that an ensemble of co-training classifiers is suitable for imbalanced datasets.

Our objective in this study was to adapt existing semi-supervised learning ensembles to datasets with high degrees of imbalance. Towards this goal, we used the approach from [11] as inspiration for two of the methods presented in this work. In [11], the authors proposed that, as the co-training sub-classifiers iterate, the balanced labeled subsets are augmented with the same instances, specifically, the most confidently labeled positive instances and the most confidently labeled negative instances. In our previous work on the problem of splice site prediction [16], we found that adding different instances to each self-training subsets leads to improved prediction because diversity is maintained. However, it was not clear what was the best way to manipulate the original distribution to ensure the largest diversity among ensemble members. Motivated by the results of our dynamic balancing technique, where only positive instances are added to the training set during the self-training iterations [15], and also by our preliminary results on ensemble approaches based on self-training classifiers [16], in the current study, we further analyze various combinations of ensembles and dynamic balancing, with focus on how the augmentation of labeled data should be managed during the semi-supervised iterations. We also experiment with co-training, in addition to self-training, and investigate how ensembles of self-training and co-training Naïve Bayes classifiers behave in the semi-supervised framework when dealing with various imbalance ratios.

A study from Wei and Dunbrack [35] that explored the effects of various distributions on supervised learning was centered around classification of human missense mutations as deleterious or neutral. By systematically varying the ratio of deleterious to neutral mutations in the training set, the authors concluded that balancing the training dataset improves the performance of SVM as evaluated by several accuracy measures, even when the distribution of the data is just mildly imbalanced. The study in [35] was performed under the assumption that the real distribution of deleterious versus neutral mutations is unknown. In the datasets used in our work [36], the proportion of true splice sites was assumed to be approximately 1% of the total number of occurrences of the "AG" dimer throughout the genome, and thus this was the highest imbalance degree that we experimented with (*i.e*., 1-to-99). However, we varied the ratio of splice site to non-splice site "AG"s from 1-to-5 to 1-to-99, to perform a systematic study of the performance obtained using ensemble-based semi-supervised approaches as a function of the imbalance ratio.

## Methods

This section describes the algorithms studied. As we focus on ensemble-based semi-supervised learning from imbalanced class distributions, specifically ensembles of self-training and co-training classifiers, we will first provide background on self-training and co-training, and also on ensemble learning. Then, we will describe the supervised ensemble approach used as a baseline in our evaluation, and finally, our proposed self-training and co-training ensemble variants.

### Self-training

Self-training, also known as self-teaching or bootstrapping, is an iterative meta-algorithm that can be wrapped around any base classifier. Yarowsky [37] originally introduced self-training and applied it to a natural language processing problem, namely word-sense disambiguation. The first step in self-training is to build a classifier using the labeled data. Then, the labeled dataset is augmented with the most confidently predicted instances from the unlabeled pool, and the model is rebuilt. The process is repeated until a criterion is met, *e.g*., until the unlabeled dataset has been fully classified or a fixed number of iterations has been reached. In our work, we classify a sub-sample of unlabeled data at each iteration (as opposed to all unlabeled data) in order to increase computation speed. The most confidently classified instances are assigned the predicted class and used to retrain the model. The remaining instances, classified with less confidence, are discarded. The algorithm iterates until the unlabeled dataset has been exhaustively sampled.

### Co-training

Blum and Mitchell [38] introduced co-training, also an iterative meta-algorithm, to solve the problem of identifying course pages among other academic web pages. Similar to self-training, co-training is applicable to any base classifier. Unlike self-training, which is a single view algorithm, co-training requires two independent and sufficient views (a.k.a., feature representations) of the same data in order to learn two classifiers. At each iteration, both classifiers label the unlabeled instances and the labeled training data of one classifier is augmented with the most confidently labeled instances predicted by the other classifier. Similar to self-training, in our work we classify only a sub-sample of unlabeled data at each iteration. Instances from the sub-sample classified with small confidence are discarded. The algorithm iterates until the unlabeled dataset has been exhaustively sampled.

### Ensembles

Ensemble learning exploits the idea that combinations of weak learners can lead to better performance. Moreover, it is known that diversity among subclassifiers is an important constraint for the success of ensemble learning [38,39]. However, learning Naïve Bayes classifiers from bootstrap replicates will not always lead to sufficiently "diverse" models, especially for problems with highly imbalanced distributions. In order to ensure sufficient variance between the original training data subsets of our highly imbalanced datasets, we used a technique initially recommended by Liu *et al*. [39], who proposed training each subclassifier of the ensemble on a balanced subset of the data, providing subclassifiers with the opportunity to learn each class equally, while the ensemble continues to reflect the original class distribution. An implementation of this technique by Li *et al*. [11] proved to be successful for the problem of sentiment classification, and was used as inspiration in our work.

### Supervised Lower Bound

Generally, supervised models trained only on the available labeled data are used as baselines for semi-supervised algorithms. Thus, the hypothesis that unlabeled data helps is verified against supervised models that entirely ignore unlabeled instances. Because our focus is on ensemble methods and ensembles of classifiers typically outperform single classifiers, the lower bound for our approaches is an ensemble of supervised classifiers. Specifically, we train ensembles of Naïve Bayes classifiers using resampled balanced subsets and use their averaged predictions to classify the test instances. This approach is referred to as the Lower Bound Ensemble (LBE).

### Ensembles inspired by the original approach: CTEO and STEO

In [11], co-training classifiers were augmented with the topmost confidently labeled positive and negative instances, as found by classifiers trained on balanced labeled subsets. The authors set the number of iterations at 50, and classified all unlabeled instances at each iteration. Moreover, the two views of the co-training classifiers were created at each iteration, using "dynamic subspace generation" (random feature splitting into two views), in order to ensure diverse subclassifiers.

However, this exact approach did not produce satisfactory results in our case, so we modified the algorithm from [11] in order to better accommodate our problem. We named the resulting approach Co-Training Ensemble inspired by the Original approach (CTEO). We also experimented with a variant where co-training was replaced with self-training, and named this variant Self-Training Ensemble inspired by the Original approach (STEO). The pseudocode for both CTEO and STEO variants is illustrated in Algorithm 1. As can be seen, Steps 7-9 are described for co-training (first line) and self-training (second line, in italic font), separately.

The first modification we made to the original ensemble-based approach, for both self-training and co-training variants, is that we kept the features fixed, *i.e*., used "static" instead of "dynamic subspace generation." For co-training, we used a nucleotide/position representation as one view, and a 3-nucleotide/position representation as the second view, under the assumption that each view is sufficient to make accurate predictions, and the views are (possibly) independent given the class.

The second modification we made is that we did not classify all unlabeled instances at each iteration; instead, we classified only a fixed subsample of the unlabeled data, as proposed in the classical co-training algorithm [38]. This alteration speeds up the computation process. The last modification that we made is that once a subsample was labeled and the top most confidently labeled instances were selected to augment the originally labeled dataset, we simply discard the rest of the subsample, thereby differing from the classical co-training approach [38] and from the original co-training ensemble approach [11]. This change also leads to faster computation times and, based on our experimentation, reduces the risk of adding mistakenly labeled instances to the labeled set in subsequent iterations. Furthermore, the last two adjustments lead to a fixed number of semi-supervised iterations, *i.e*., as the algorithm ends when the unlabeled data pool is exhausted. We use a subsample size that is dependent on the dataset size, and selected such that the algorithm iterates approximately the same number of times (50) for each set of experiments, for a certain imbalance degree. After the iterations terminate, the ensemble is used to classify the test set by averaging the predictions of the constituent subclassifiers.

An important observation regarding Step 9 in Algorithm 1 is that, in the case of co-training, when the two classifiers based on *view*1 and *view*2, respectively, make their predictions, an instance is added to the pseudolabeled set *P *only if (1) no conflict exists between the classifiers, *i.e.*, both classifiers agree on the label, and (2) one classifier predicts the label with high confidence, while the other predicts the same label with low confidence. These conditions ensure that the two views inform each other of their best predictions, thereby enhancing each other's learning.

**Algorithm 1 **Ensembles inspired by the original approach [11] - CTEO/STEO

1: Given: a training set comprised of labeled and unlabeled data *D *= (*D_l_, D_u_*), *|D_l_| ≪ |D_u_|*

2: Create *U *by picking *S *random instances from *D_u _*and update *D_u _*= *D_u _*- *U , S *= sample size

3: Generate *N *balanced subsets from *D_l _*: *D*_*l*1_, . . ., *D_ln_*

4: **repeat**

5:    Initialize *P *= *∅*

6:    **for ***i *= 1 to *N ***do**

7:       CT: Train subclassifiers *C*_*i*1 _on *view*_1 _and *C*_*i*2 _on *view*_2 _of balanced subset *D_li_*

        *ST: Train subclassifier C_i _on combined views of balanced subset D_li_*

8:       CT: Classify instances in *U *using the classifiers *C*_*i*1 _and *C*_*i*2_

        *ST: Classify instances in U using subclassifier C_i_*

9:       CT: Use *C*_*i*1 _and *C*_*i*2 _to select 2 positive and 2 negative instances and add them to *P*

        *ST: Use C_i _to select 2 positive and 2 negative instances, and add them to P*

10:    **end for**

11:    Augment each balanced subset with the instances from *P*

12:    Discard remaining unused instances from *U*

13:    Create a new unlabeled sample *U *and update *D_u _*= *D_u _*- *U*

14: **until ***U *is empty (*i.e*., the unlabeled data is exhausted)

As mentioned above, STEO differs from the co-training based ensemble, CTEO, at Steps 7-9 in Algorithm 1: instead of using two subclassifiers trained on two different views, only one classifier is built using all features (*view*_1 _and *view*_2 _combined), and then this classifier is used to select the best two positive predictions and the best two negative predictions. Because each subclassifier in CTEO contributes one positive and one negative instance, after one iteration, the set *P *of pseudo-labeled instances contains 2N positive instances and 2N negative instances. Therefore, in STEO, we add the top two positives and top two negatives as predicted by the same subclassifier *C_i _*in order to maintain an augmentation rate identical to the augmentation rate in CTEO. After the semi-supervised iterations terminate, the ensemble is used to predict the labels of the test set. The predictions of every subclassifier in the ensemble on a test instance are combined via averaging, and the resulting probabilities represent the final class distribution of the instance.

### Ensembles using dynamic balancing with positive: STEP and CTEP

The following two approaches use the dynamic balancing technique proposed in [15], found to be successful for the classical self-training algorithm when the dataset exhibits imbalanced distributions. The dynamic balancing occurs during the semi-supervised iterations of the algorithm and uses only the instances that the classifier (or subclassifiers in the ensemble) predicted as positive to augment the originally labeled set. In the ensemble context, subclassifiers are used to select the most confidently predicted positive instances. These variants are named Co-Training Ensemble with Positive (CTEP) and Self-Training Ensemble with Positive (STEP), and illustrated in Algorithm 2. As before, the co-training and self-training variants differ at Steps 7-9. For CTEP, during Step 9, the instance classified as positive with topmost confidence in one view and low confidence in the second view is added to *P*, and vice-versa. For STEP, the two most confidently labeled positive instances are added to *P*, such that the augmentation rate is identical to that from CTEP.

**Algorithm 2 **Ensembles using dynamic balancing with positive - STEP/CTEP

1: Given: a training set comprised of labeled and unlabeled data *D *= (*D_l_, D_u_*), *|D_l_| ≪ |D_u_|*

2: Create *U *by picking *S *random instances from *D_u _*and update *D_u _*= *D_u _*- *U , S *= sample size

3: Generate *N *balanced subsets from *D_l _*: *D*_*l*1_, . . ., *D_ln_*

4: **repeat**

5:     Initialize *P *= *∅*

6:     **for ***i *= 1 to *N ***do**

7:         CT: Train subclassifiers *C*_*i*1 _on *view*_1 _and *C*_*i*2 _on *view*_2 _of balanced subset *D_li_*

            *ST: Train subclassifier C_i _on combined views of balanced subset D_li_*

8:         CT: Classify instances in *U *using subclassifiers *C*_*i*1 _and *C*_*i*2_

            *ST: Classify instances in U using subclassifier C_i_*

9:         CT: Use *C*_*i*1 _and *C*_*i*2 _to select 2 positive instances and add them to *P*

            *ST: Use C_i _to select 2 positive instances and add them to P*

10:     **end for**

11:     Augment each balanced subset with the instances from *P*

12:     Discard remaining unused instances from *U*

13:     Create a new unlabeled sample *U *and update *Du *= *Du *- *U*

14: **until ***U *is empty (*i.e*., the unlabeled data is exhausted)

### Ensembles that distribute the newly labeled instances: CTEOD and STEOD

Our next semi-supervised ensemble variants are based on CTEO and STEO, respectively, and distribute the most confidently labeled instances among the classifiers in the ensemble. They are referred to as Co-Training Ensemble Original Distributed (CTEOD) and Self-Training Ensemble Original Distributed (STEOD), and shown in Algorithm 3. In CTEOD and STEOD, as opposed to CTEO and STEO, instances are distributed such that each balanced subset receives two unique instances, one positive and one negative, from each view, instead of adding all instances from *P *to every balanced subset. The idea that motivated this change was that different instance distributions would ensure a certain level of diversity for the constituent classifiers of the ensemble. In Algorithm 3, the co-training and self-training variants differ at Steps 6-8. As can be seen, the main difference compared to CTEO and STEO is at Step 9, where classifier *C*_*i*1 _trained on *view*_1 _is augmented with the top positive and top negative instances as predicted by classifier *C*_*i*2 _trained on *view*_2_, and vice-versa. Therefore, each balanced subset is augmented with two positive instances and two negative instances, and the ensemble better conserves its initial diversity.

**Algorithm 3 **Ensembles that distribute newly labeled instances - CTEOD/STEOD

1: Given: a training set comprised of labeled and unlabeled data *D *= (*D_l_, D_u_*), *|D_l_| ≪ |D_u_|*

2: Create *U *by picking *S *random instances from *Du *and update *D_u _*= *D_u _*- *U , S *= sample size

3: Generate *N *balanced subsets from *D_l _*: *D*_*l*1_, . . ., *D_ln_*

4: **repeat**

5:     **for ***i *= 1 to *N ***do**

6:         CT: Train subclassifiers *C*_*i*1 _on *view*_1 _and *C*_*i*2 _on *view*_2 _of balanced subset *D_li_*

            *ST: Train subclassifier C_i _on combined views of balanced subset D_li_*

7:         CT: Classify instances in *U *using subclassifiers *C*_*i*1 _and *C*_*i*2_

            *ST: Classify instances in U using subclassifier C_i_*

8:         CT: Use *C*_*i*1 _and *C*_*i*2 _to select 2 positive instances and 2 negative instances

            *ST: Use C_i _to select 2 positive instances and 2 negative instances*

9:     Augment current balanced subset, *D_li_*, with selected positive and negative instances

10:     **end for**

11:     Discard remaining unused instances from *U*

12:     Create a new unlabeled sample *U *and update *D_u _*= *D_u _*- *U*

13: **until ***U *is empty (*i.e*., the unlabeled data is exhausted)

### Ensembles that distribute only positive instances - CTEPD and STEPD

Our last semi-supervised ensemble variants are based on CTEP and STEP. We again use the dynamic balancing technique from [15] that adds only positive instances in the semi-supervised iterations. In addition, instances are distributed among the balanced labeled subsets, such that diversity is maintained and the subclassifiers are trained on diverse enough instance subsets, thus increasing the diversity of the constituent ensemble classifiers. The resulting variants are named Co-Training Ensemble with Positive Distributed (CTEPD) and Self-Training Ensemble with Positive Distributed (STEPD), and shown in Algorithm 4. The co-training and self-training variants differ at Steps 6-8. Overall, at each iteration, 2N unique positive instances augment the ensemble in which N is the imbalance degree since two instances originated from each co-training subclassifier. More specifically, each of the N subclassifier receives two positive instances, different from the instances received by the other subclassifiers.

## Data and feature representation

For our empirical evaluation, we used five imbalanced and relatively large datasets, originally published in [36] and used for a domain adaptation study. The datasets belong to five organisms, *C. elegans*, which contains approximately 120K instances, and *C. remanei, P. pacificus, D. melanogaster*, and *A. thaliana*, which contain approximately 160K instances each. In each of these datasets, the true acceptor splice sites represent 1% of the total number of instances, hence the datasets exhibit a 1-to-99 imbalance ratio. The class label of each instance is either positive to indicate a true acceptor splice site, or negative to indicate a decoy splice site.

**Algorithm 4 **Ensembles that distribute only positive instances - CTEPD/STEPD

1: Given: a training set comprised of labeled and unlabeled data *D *= (*D_l_, D_u_*), *|D_l_| ≪ |D_u_|*

2: Create *U *by picking *S *random instances from *D_u _*and update *D_u _*= *D_u _*- *U , S *= sample size

3: Generate *N *balanced subsets from *D_l _*: *D_l_*_1_, . . ., *D_ln_*

4: **repeat**

5:     **for ***i *= 1 to *N ***do**

6:         CT: Train subclassifiers *C*_*i*1 _on *view*_1 _and *C*_*i*2 _on *view*_2 _of balanced subset *D_li_*

            *ST: Train subclassifier C_i _on combined views of balanced subset D_li_*

7:         CT: Classify instances in *U *using subclassifiers *C*_*i*1 _and *C*_*i*2_

            *ST: Classify instances in U using subclassifier C_i_*

8:         CT: Use *C*_*i*1 _and *C*_*i*2 _to select 2 positive instances and add them to *P*

            *ST: Use C_i _to select 2 positive instances and add them to P*

9:         Augment the current balanced subset with positive and negative instances

10:     **end for**

11:     Discard remaining unused instances from *U*

12:     Create a new unlabeled sample *U *and update *D_u _*= *D_u _*- *U*

13: **until ***U *is empty (*i.e*., the unlabeled data is exhausted)

**Figure 1:**
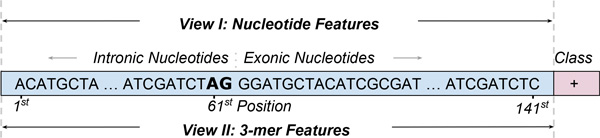
**Acceptor Splice Site: Each instance is a 141-nt window around the splice site, with the "AG" dimer starting at position 61**. The sequence is used to generate two views for co-training: one based on nucleotides and another one based on 3-mers.

In our previous work [15,16], we used 141-dimensional feature vectors to represent instances, $x=\left(x_{1},x_{2},...,x_{N}\right)\in {\mathbb{R}}^{N}$ (*N *= 141). Each dimension corresponds to a position in the original sequences, and takes as values one of the four nucleotides *{A, C, G, T }*, as shown in Figure 1. Specifically, feature *x_i _*indicates the nucleotide found at the corresponding position *i*. In the current work, because the co-training algorithm requires two views of the data, we use the nucleotide/position representation as the first view and the 3-nucleotide/position representation from [40] as the second view. As the name suggests, 3-nucleotides are sequences of length 3 (also referred to as 3-mers or "codons"). Intuitively, 3-nucleotides can capture more context information, as compared to single nucleotides. The 3-nucleotide/position representation, thus, captures additional correlations between nucleotides, while maintaining a low number of features (specifically, 139 features for our sequences which have length 141), thereby making the two views comparable. Given that nucleotide/position and 3-nucleotide/position features have shown to be effective in a domain adaptation scenario [40], we hypothesize that semi-supervised learning could also benefit from these feature representations. For self-training, we used the two views together and trained the classifiers on the complete set of features.

## 1 Experimental setup

### 1.1 Research questions

The experiments were designed to answer the following research questions:

1 Which ensembles are more affected by imbalanced distributions, supervised ensembles or semi-supervised ensembles?

2 How does the performance of the approaches vary with the imbalance degree?

3 What is the best strategy for utilizing newly labeled instances when using ensembles of semi-supervised classifiers trained on highly imbalanced data?

The five datasets used in this study were labeled, and therefore we were able to create, via resampling, various data subsets with various imbalance degrees (from 1-to-5 to the original 1-to-99), in order to observe the algorithms' performance with respect to the imbalance degree. For example, in the original *D. melanogaster *dataset, with the imbalance degree of 1-to-99, there are 159,748 instances, 1,598 positives and 158,150 negatives. In order to create the dataset for each experiment, we kept the positive instances and resampled at random *N *number of negative instances to obtain a new dataset with an imbalance degree of 1-to-N. For example, in the 1-to-5 experimental dataset for *D. melanogaster*, there are 9,588 instances, 1,598 positives and 7,990 negatives. The rest of the datasets, corresponding to higher imbalance degrees, were built incrementally so that the dataset with the imbalance degree of 1-to-10 contains all the instances from the 1-to-5 dataset, and also contains additional negative instances to reach the desired imbalance.

As can be seen, for each experiment, the number of instances varies, and in the semi-supervised iterations, we used a sample size proportional to the dataset size, such that the experiments iterate roughly the same number of times.

Because classifiers are highly susceptible to data variation and prone to sampling bias, we evaluated the models using 10-fold cross validation in which nine folds were used to train the model and the tenth fold was used for testing. Data comprising the nine training folds is further divided into labeled and unlabeled. We randomly pick labeled instances such that the ratio of positive to negative is maintained and the total number of instances represents no more than 1%.

### Evaluation

Because of the highly skewed distributions of the datasets, in order to objectively measure the predictive ability of our approaches, we compared their performance in terms of the area under the Precision-Recall Curve (auPRC), which is a more appropriate assessment measure than the area under the Receiver-Operating Curve (auROC) [41,42]. In order to evaluate the results, we averaged auPRC values for the minority (positive) class across the ten folds for each organism. While the trends are generally maintained for individual organisms, we report averages of auPRC values over the five organisms, for easier interpretation. We performed two-tailed paired t-tests, as opposed to one-tailed t-tests, to identify statistically significant differences in either direction, on all semi-supervised algorithms for all variations of imbalance degrees. The test determines if the difference between a semi-supervised ensemble algorithm and its corresponding supervised ensemble baseline (seen as a lower bound) is statistically significant [43].

## Results and discussion

Our experimental results are compiled in Table 1. The first column represents the imbalance degree of the experiment, which is varied from 1-to-5 to 1-to-99, by randomly discarding negative (majority) instances. The second column, LBE, shows the results of the supervised lower bound, which is also an ensemble, consisting of supervised classifiers. LBE is used as the baseline against which to compare the semi-supervised approaches. From the third column onwards, each method is presented for co-training and self-training. The results are discussed by addressing the research questions. Values marked with bold font represent performances of the semi-supervised experiments that outperform the supervised lower bound. The starred (*) values denote experiments whose variation in comparison to the lower bound was found to be statistically significant by the paired t-test in all five organisms. The values marked with a plus (†) indicate experiments that the paired t-test found to be statistically significant in four out of five organisms. The values marked with a diamond (◇) indicate experiments that the paired t-test found to be statistically significant in three out of five organisms.

**Table 1:** Table of Results.

**Imbal**. Degree	LBE	CTEO	STEO	CTEP	STEP	CTEOD	STEOD	CTEPD	STEPD
1-to-5	0.452	**0.526**^◇^	0.567*	0.647*	**0.479**^◇^	0.692*	0.652*	**0.644**†	**0.612**^◇^
1-to-10	0.434	**0.462**	**0.455**†	**0.557**†	0.343†	0.584*	**0.573**†	**0.584**†	**0.573**†
1-to-20	0.437	0.434	**0.440**^◇^	**0.522**†	0.292^◇^	**0.515**^◇^	**0.529**†	**0.523**^◇^	0.526*
1-to-25	0.437	0.384^◇^	0.423^◇^	**0.497**^◇^	0.245*	**0.507**^◇^	**0.465**^◇^	**0.510**^◇^	**0.507**†
1-to-30	0.430	0.336*	0.408^◇^	**0.484**^◇^	0.239*	**0.509**†	**0.470**^◇^	**0.503**^◇^	0.514*
1-to-40	0.443	0.404†	0.409	**0.492**^◇^	0.222†	**0.503**^◇^	**0.468**	**0.504**^◇^	**0.497**†
1-to-50	0.450	0.372†	0.409^◇^	**0.491**	0.236*	**0.508**^◇^	**0.451**	**0.504**	**0.486**
1-to-60	0.471	0.388†	0.398	**0.472**	0.195†	**0.496**	0.423	**0.494**^◇^	**0.474**
1-to-70	0.450	0.392†	0.411	**0.462**	0.207†	**0.474**^◇^	0.444	**0.480**^◇^	**0.478**
1-to-75	0.454	0.388	0.399^◇^	**0.460**^◇^	0.249†	**0.483**^◇^	0.435	**0.483**	**0.471**
1-to-80	0.449	0.353†	0.386†	0.436	0.204*	**0.457**	0.421^◇^	**0.460**^◇^	**0.465**†
1-to-90	0.453	0.359†	0.410	0.449	0.242	**0.470**	0.423	**0.473**†	**0.456**
1-to-99	0.446	0.376	0.389^◇^	0.440†	0.226†	**0.464**	0.414	**0.459**	**0.457**

The values represent averages of auPRC values for the positive class over the five organisms when the class imbalance degree varies from 1-to-5 to 1-to-99 and the amount of labeled instances represents less than 1% of the training data. LBE is the ensemble-based supervised lower bound. CTEO and STEO are the co-training-based and self-training-based ensembles inspired by the original approach in [11]. CTEP and STEP are the co-training and self-training based ensembles that use the "dynamic balancing" approach introduced in [15], in which only positive instances are used in semi-supervised iterations to augment the originally labeled training data. CTEOD and STEOD add positive and negative instances but distribute them among all subclassifiers, such that the balance and diversity of each subclassifier's labeled subset is maintained. CTEPD and STEPD use "dynamic balancing" but also distribute instances among all subclassifiers. The bold font denotes the semi-supervised experiments that outperform the lower bound. The starred (*) values denote experiments whose variation in comparison to the lower bound was found to be statistically significant by the paired t-test in all five organisms. The values marked with a plus (†) indicate experiments that the paired t-test found to be statistically significant in four out of five organisms. The values marked with a diamond (◇) indicate experiments that the paired t-test found to be statistically significant in three out of five organisms.

1 *Which ensembles are more affected by imbalanced distributions, supervised ensembles or semi-supervised ensembles? *The supervised baseline remains somewhat constant irrespective of the imbalance degree, showing that additional labeled data can help alleviate problems caused by extreme cases of imbalance. Note that experiments with milder degrees of imbalance contain less instances than experiments with higher degrees of imbalance, given the way we constructed our datasets. When the imbalance degree is the highest, 1-to-99, we used the entire dataset. Compared to supervised learning, semi-supervised learning ensembles show a slow decrease in performance as the imbalance degrees become more prominent, most probably due to the fact that additional unlabeled data is more difficult to label correctly.

2 *How does the performance of the approaches vary with the imbalance degree? *As can be seen from the table, for lower degrees of imbalance (1-to-5 to 1-to-40), semi-supervised ensembles are considerably surpassing the supervised baselines. As the experiments become increasingly difficult (the imbalance degree becomes more prominent), some semi-supervised ensembles deteriorate as a result of unlabeled data being incorrectly classified with high confidence, and they are surpassed by the supervised baselines.

In the original study [11] that inspired our CTEO and STEO variants, the ensemble approach was used to predict the sentiment polarity of Amazon reviews with imbalance degrees ranging between 1-to-5 and 1-to-8, and proved to be superior to supervised baselines. Our variants, CTEO and STEO, also produced good results for experiments with relatively low imbalance degrees, 1-to-5 and 1-to-10. From 1-to-20 onwards, however, the CTEO and STEO semi-supervised ensembles performed worse than their supervised baselines, but, surprisingly, the self-training ensembles more effectively utilized the unlabeled data as compared to the co-training ensembles. For approaches that employ the "dynamic balancing" technique [39] in which only positive instances are used, the ensemble based on co-training CTEP leveraged the unlabeled data and surpassed the supervised counterpart for experiments with up to 1-to-60 imbalance degree, after which point no discernible difference was observed between CTEP and the baseline. The ensemble based on self-training, STEP, is more sensitive and was deteriorated by the unlabeled data beginning with Experiment 1-to-10. The "pseudo" positive instances could have been misclassified, thereby misleading the classifiers, which all use the same newly labeled positive instances. In general, the ensembles that do not distribute the instances among their subclassifiers deteriorate and fall below the baseline for moderate and high degrees of imbalance. Variants of the algorithms where instances are distributed tend to outperform the other approaches. When both positive and negative instances are used to augment the labeled data, CTEOD and STEOD outperformed the not-distributed versions CTEO and STEO. The self-training based approach STEOD still falls below the supervised baseline for experiments over 1-to-50, but the co-training based approach CTEOD is surpassing the baseline for all experiments. The variants CTEPD and STEPD, which add only positive instances and distribute them, surpassed the baseline for all experiments. No significant difference in performance between CTEOD and CTEPD was observed, but STEPD out-performed STEOD and surpassed the baseline in all experiments. Thus, the "dynamic" balancing approach proved to be more useful for the self-training based ensemble.

3 *What is the best strategy for utilizing newly labeled instances when using ensembles of semi-supervised classifiers trained on highly imbalanced data? *One important observation that can be made based on our results is that the distribution of the newly labeled instances among subclassifiers in order to ensure subclassifier diversity is a useful approach for semi-supervised ensembles. Variants that distribute the newly labeled instances (either positive and negative for CTEOD and STEOD, or solely positive for CTEPD and STEPD) achieved overall better performance than the classifiers that receive all the newly labeled instances (CTEO, STEO, CTEP, and STEP). Therefore, the conclusion is that diversity in this case is more useful than the addition of substantially more "pseudo" (newly) labeled instances during the semi-supervised iterations.

Our results for the paired t-test showed no particular consistency, specifically some experiments and results were statistically significant and others were not.

## Conclusions

In this work, we proposed and studied several ensemble-based variants of two popular semi-supervised learning algorithms, self-training and co-training, and tested their performance on the task of predicting splice sites. The task was formulated as a binary classification problem and the models' performance was tested on five large acceptor splice site datasets from five organisms. We adapted the ensembles to address the highly imbalanced datasets of our case study, and we used various approaches to augment the labeled data during the semi-supervised iterations. Our results showed that one important constraint of any ensemble (based on self-training or co-training) is to maintain diversity of the ensemble's subclassifiers, by augmenting the labeled subsets of subclassifiers with unique newly labeled instances. Maintaining the ensemble diversity by adding less but unique instances to each sub-classifier is a better approach than adding the same (larger sets of) instances to all subclassifiers.

In order to address highly skewed distributions, we found that dynamically balancing of ensembles by utilizing only positive instances during semi-supervised iterations to augment the labeled data and distributing them among constituent subclassifiers is a useful technique that benefits both types of ensembles, but especially the self-training-based approaches. For co-training-based approaches, whether instances from both classes are added (CTEOD) or just positives (CTEPD), the performance variations are negligible. Both approaches CTEPD and CTEOD surpass the other semi-supervised ensembles studied.

In general, our results show that ensembles based on self-training are surpassed by the ensembles based on co-training, a trend that has been reported many times in the literature for single classifiers, *e.g*., in the prediction of alternatively spliced exons [3], or text classification [5].

As part of future work, we consider exploring other base learners (*e.g*., large margin classifiers) for self-training and co-training algorithms. Given that aggregated stacking produced the best results for protein function prediction and genetic interactions prediction in [44], it would be interesting to explore meta-learning and ensemble selection for the splice site prediction problem. Transductive approaches demonstrated great potential for protein classification from imbalanced datasets [32], and SVM has previously been shown to successfully identify splice sites [18]. Therefore, the behavior of SVM in a transductive context is of interest in relation to splice site prediction.

## Competing interests

The authors declare that they have no competing interests.

## Authors' contributions

A.S. and D.C. designed the study. A.S. carried out the computational aspect of the analysis. All authors participated in the writing of the manuscript; all authors read and approved the final manuscript.

## Declarations

The authors gratefully acknowledge financial support for the publication of this article from the Department of Computing and Information Sciences at Kansas State University.

This article has been published as part of *BMC Systems Biology *Volume 9 Supplement 5, 2015: Selected articles from the IEE International Conference on Bioinformatics and Biomedicine (BIBM 2014): Bioinformatics. The full contents of the supplement are available online at http://www.biomedcentral.com/bmcsystbiol/supplements/9/S5.
